# Screening and verification of antiviral compounds against HSV-1 using a method based on a plaque inhibition assay

**DOI:** 10.1186/s12879-023-08843-3

**Published:** 2023-12-19

**Authors:** Yingxian Yin, Jiahui Li, Ling Su, Zhiying Ou, Qingqun Lv, Misi Xiao, Changbing Wang, Dan Zeng, Yiling Gu, Fengxia Yang, Minxia Chen, Shaojuan Feng, Wanming Hu, Fengling Bu, Bing Zhu, Yi Xu

**Affiliations:** 1 Guangzhou Institute of Pediatrics, Guangzhou Women and Children’s Medical Center, Guangzhou Medical University, Guangzhou, 510623 China; 2 Department of Infectious Diseases, Guangzhou Women and Children’s Medical Center, Guangzhou Medical University, Guangzhou, 510120 China; 3 Department of Genetics and Endocrinology, Guangzhou Women and Children’s Medical Center, Guangzhou Medical University, Guangzhou, 510623 China; 4 School of Pediatrics, Guangzhou Medical University, Guangzhou, 510623 China; 5 Central Laboratory, Guangzhou Institute of Pediatrics, Guangzhou Women and Children’s Medical Center, Guangzhou Medical University, Guangzhou, 510120 China; 6 Department of Oral and Maxillofacial Surgery, Women and Children’s Medical Center, Guangzhou Medical University, Guangzhou, 510120 China; 7 Guangzhou Women and Children’s Medical Center, Guangzhou Medical University, Operating room, Guangzhou, 510120 China; 8 Department of Disease Control and Prevention, Guangzhou Women and Children’s Medical Center, Guangzhou Medical University, Guangzhou, 510120 China

**Keywords:** Antiviral drug screening, HSV-1, Plaque formation, Plaque inhibition test

## Abstract

**Background:**

Herpes simplex virus type 1 (HSV-1) infection is a common viral disease that mainly causes oral lesions, but can also cause genital lesions in some instances. Current treatments with nucleoside analogs are limited by the emergence of drug resistance. Therefore, novel anti-HSV-1 drugs are urgently needed.

**Methods:**

In this study, we screened a library of 2080 compounds for anti-HSV-1 activity using a plaque formation assay. We selected 11 potential inhibitors of HSV-1 and further evaluated their antiviral effects by plaque reduction assay and real-time polymerase chain reaction (qPCR).

**Results:**

Five compounds, namely ginsenoside Rd, brassinolide, rosamultin, 3’-hydroxy puerarin, and clinafloxacin HCl, showed potent anti-HSV-1 activity and completely suppressed plaque formation at a concentration of 10 µM. Among them, clinafloxacin HCl, a fluoroquinolone antibiotic, exhibited a high selectivity index for HSV-1.

**Conclusions:**

Our findings suggest that these five compounds have potential antiviral properties against HSV-1 and may have different mechanisms of action. Further studies are warranted to elucidate the antiviral mechanisms of these compounds and to explore their therapeutic potential for HSV-1 infection.

**Supplementary Information:**

The online version contains supplementary material available at 10.1186/s12879-023-08843-3.

## Introduction

In children, HSV-1 is a common cause of mucosal diseases, including orofacial infections, keratitis, and encephalitis, which can lead to morbidity and mortality [1, 2]. Keratitis caused by HSV-1 infection usually results in corneal scarring and loss of vision [3]. Disseminated HSV-1 infection in the newborn will lead to neurodevelopmental disorders [4]. Increasing evidence suggests that HSV-1 is becoming an important infectious risk factor for neonatal and immunocompromised patients [5–7]. Stress may increase the susceptibility to primary HSV-1 infection and reactivate the latent virus in neurons [8]. Acyclovir is the most effective antiviral drug for HSV infections, as it has high selectivity, bioavailability, and safety [9]. This drug belongs to the class of nucleoside analogues, which are widely used for treating HSV infection [10, 11]. Drug resistance is a major concern when treating HSV-1 infections with nucleoside analogs, such as acyclovir. These drugs cannot prevent HSV-1 from establishing latency in neurons, which poses a major clinical challenge [10]. Moreover, immunosuppressed patients who undergo prolonged treatment may develop acyclovir-resistant strains [12, 13].

Drug screening is a crucial approach for identifying novel antiviral inhibitors. Most antiviral drug screenings for HSV focused on a functional target of the viral particle. Virtual screening using molecular docking of inhibitors and cyclin-dependent kinase 2 (CDK2) has been reported [14]. Infected cell protein 0 (ICP0 protein) of HSV has been used to screen inhibitors blocking its E3 Ubiquitin ligase activity in an in vitro screening assay [15].

Previously, an improved plaque assay-based high-throughput antiviral drug screening method has been documented [16]. In the current study, an antiviral drug screening was performed using a HSV-1/Vero system based on an improved plaque assay, screening 11 potential inhibitors from a library of 2080 compounds. This method based on a single infectious viral particle can theoretically help discover novel antiviral inhibitors associated with different viral life cycle stages including attachment, entry, replication, maturation and release.

## Materials and methods

### Cell cultures and viruses

We cultured Vero cells (CCL-81, ATCC) and SK-N-SH cells (HTB11, ATCC) in DMEM supplemented with 10% fetal bovine serum. To obtain the HSV-1 strain GZ21P2, which is sensitive to acyclovir, we isolated it from a clinical sample and purified it by selecting a single plaque under agarose overlay medium. We then propagated the purified viral stock of GZ21P2 in Vero cells and assessed its titers using the plaque formation method.

### Drug preparation

A library of 2,080 unique compounds (Table S1) was provided to us by Selleck (Shanghai) in 30 µL tubes of 10 mM DMSO. The verification compounds were dissolved in DMSO as per the manufacturer’s instructions. Subsequently, stock solutions of 100 mM or lower were prepared and diluted with DMEM to obtain different concentrations.

### Drug screening

We screened a library of 10 mM compounds for anti-HSV-1 activity using Vero cells infected with HSV-1 strain GZ21P2. We added 1.6 µL of each compound to the overlay medium containing 1.2% RC-591 (FMC Polymer, USA) in 96-well plates (two replicates per well). We used acyclovir (100 µM) as a positive control and DMSO as a negative control (four wells each). The final concentration of DMSO in each well was 1% (v/v). After four days, we fixed and stained the cells with formaldehyde (8%) and neutral red (0.3%). We considered the experiment valid if the positive control wells had no plaques and the negative control wells had plaques. We selected the compounds that prevented plaque formation (intact cell monolayer) as potential inhibitors. We tested these inhibitors again at 100 µM, 50 µM, and 10 µM in a second round using the same procedure. The drugs that completely inhibited plaque formation at 10 µM were effective candidate drugs for the following inhibition effect verification. We followed our previously published method with minor modifications for this part [16].

### CCK-8 cytotoxicity assay

In brief, SN-N-SH cells were initially seeded into 96-well plates at a density of 1 × 10^4^ cells per well and incubated at 37 ℃ in a 5% CO_2_ incubator. After a 24-hour incubation, 100 µL of medium containing different concentration of drugs were added to each well, replacing the previous medium. We also used DMSO as a zero-concentration negative control. The final concentration of DMSO in each well was 0.1% (v/v). Following another 48-hour incubation period, the culture medium in each well was again replaced with 100 µL of fresh medium containing 10 µL of CCK-8 solution (Dojindo). After a further two-hour incubation, the plates were subjected to absorbance measurement at 450 nm using a microplate reader. The 50% cytotoxic concentration (CC_50_) of each compound was estimated by performing a linear regression analysis. We followed the manufacturer’s protocol for the CCK-8 solution.

### Plaque reduction assay

To assess the anti-HSV-1 effect of these compounds against HSV-1, Vero cells were grown in 24-well plates (5 × 10^4^ cells/well). After 24 h, the cells were infected with HSV-1 at a multiplicity of infection (MOI) of 0.1 for 1 h, and then the supernatant was removed and replaced with fresh DMEM containing various concentrations of the compounds (DMSO as a negative control with zero drug concentration). The final concentration of DMSO in each well is 0.1% (v/v). After 24 h of incubation, the cells were lysed by three cycles of freeze-thaw, and the supernatant was collected by centrifugation at 5, 000 rpm. The supernatant was serially diluted, and 60 µL of the dilutions was added to Vero cell monolayers in 96-well plates. After 1 h of incubation at 37℃, an overlay medium (100 µL) containing 1.2% RC-591 was added directly to each well. The cells were then fixed and stained as described in the drug screening section. The number of plaques was counted, and viral inhibition percentage was calculated using the following formula: viral inhibition (%) = [1-(number of plaques) _inhibitor_/ (number of plaques) _control_] ×100 [17]. The half-maximal inhibitory concentration (IC50) was determined using Graphpad Prism 8.0.

### Quantification of viral DNA

To evaluate the antiviral effects of candidate compounds based on the viral DNA concentration, we performed the following steps. SN-N-SH cells seeded in 24-well plates a density of 4 × 10^4^ cells per well. The cells were pretreated with the different concentrations of the candidate compounds for 24 hours, followed by addition of viral stock (0.1 MOI) to each well for 1 hour to allow infection. Subsequently, the supernatant was removed and cells were cultured with drug-containing medium at 37℃ for an additional 24 hours. DMSO was used as a negative control with zero drug concentration. The final concentration of DMSO in each well is 0.1% (v/v). The viral DNA was extracted from the supernatant using the TIANamp Virus DNA/RNA Kit (Tiangen) and analyzed by qPCR. The primer pairs targeting the pol gene of HSV-1 (PL 5’-ATCAACTTCGACTGGCCCTTC-3’, and PR, 5’-CCGTACATGTCGATGTTCACC-3’) were previously described by Lakeman and Whitley [18].

### Statistical analysis

The data are expressed as the Mean ± SD and analyzed using an unpaired Student’s t-test. The level of significance was set at p < 0.05, p < 0.01, P < 0.001 to determine the statistical significance of the results.

## Results

### Screening of a unique compound library for inhibitors of HSV-1

**Fig. 1:**
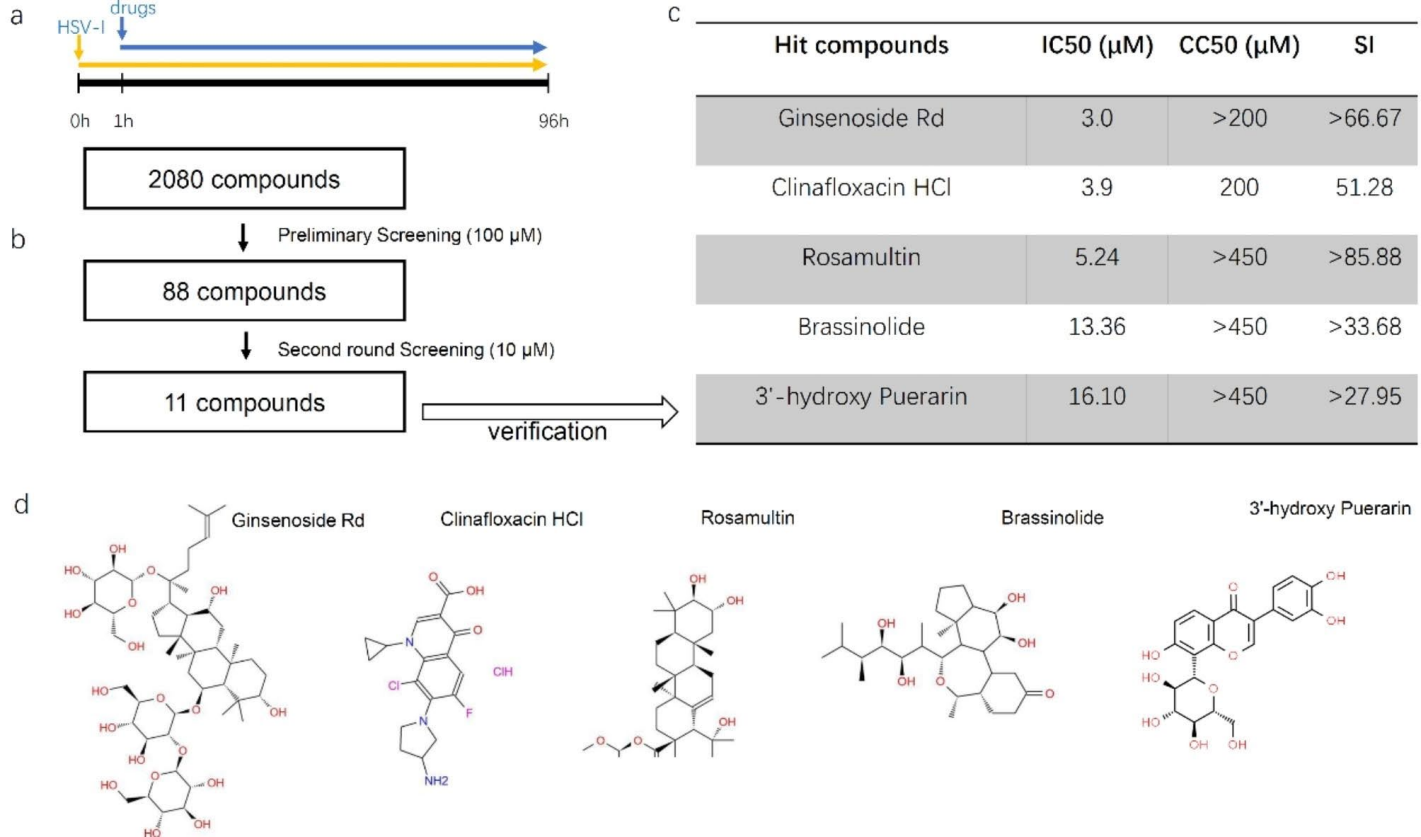
Antiviral drug screening for inhibitors of HSV-1 from a 2080-compound library. (a) Timeline of drug screening. b) Flowchart for drug screening. (c) Verification of five inhibitors. This result is based on plaque reduction effects of candidate inhibitors and their cytotoxicity against SK-N-SH cells. (d) Chemical structures of five candidate inhibitors

The diagram in Fig. 1 outlines the screening process used in this study (Fig. 1). In the initial screening phase, 88 compounds were evaluated at a concentration of 100 µM. These compounds demonstrated complete inhibition of plaque formation, whereas the remaining compounds in the drug library did not exhibit this effect. Additionally, those compounds that exhibited cytotoxic activity against Vero cells completely caused cellular detachment at the bottom of wells in 96-well plates. From the initial screening, 11 candidate inhibitors were selected in the second round of screening. These compounds were found to completely suppress plaque formation at a concentration of 10 µM (Fig. 1).

### Cytotoxicity

**Fig. 2:**
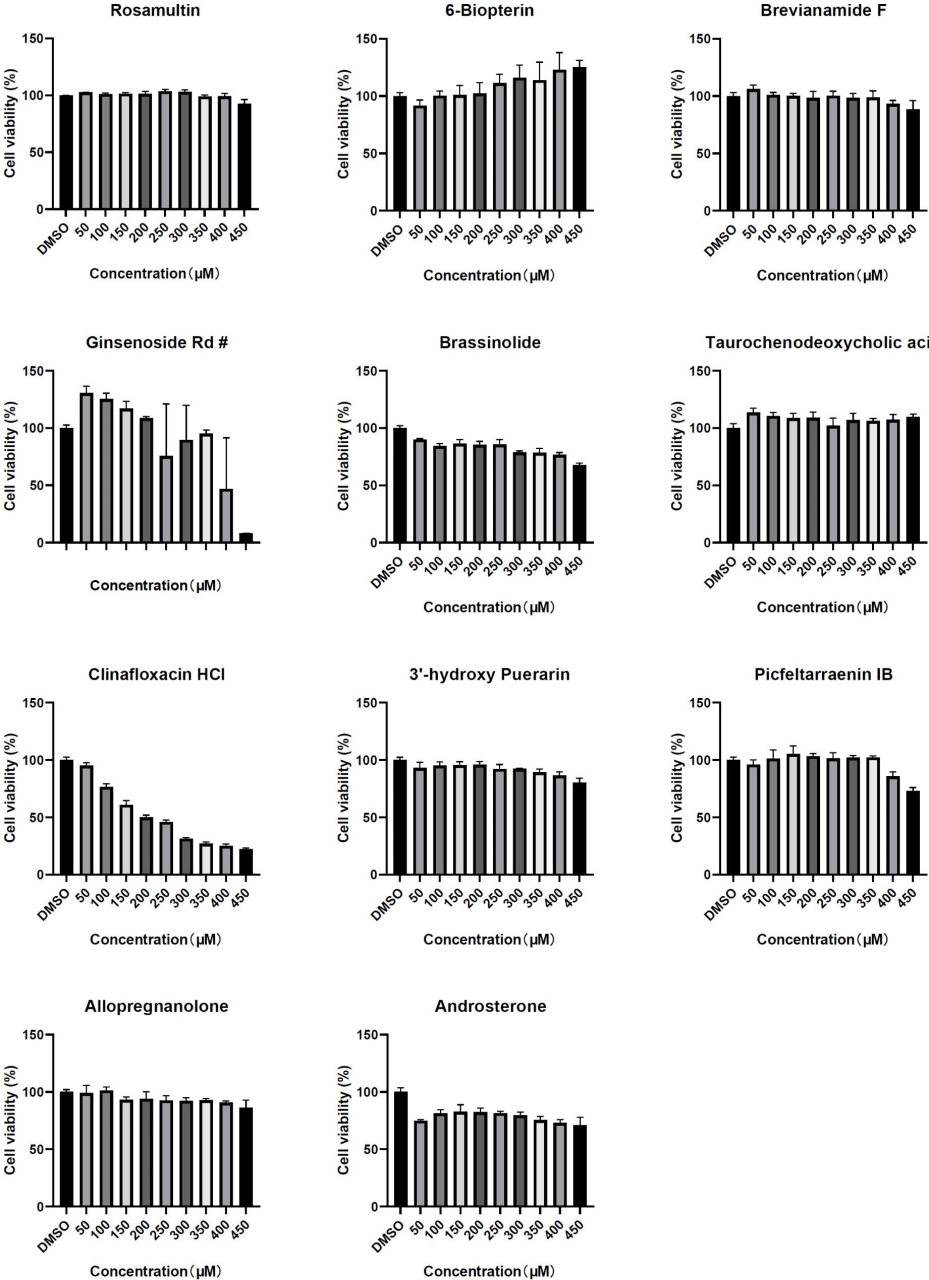
Cell viability of 11 candidate inhibitors of HSV-1. After adding candidate inhibitors with different concentrations, the viability of SK-N-SH cells was measured by a cell counting kit (CCK-8)

The majority of the 11 compounds evaluated in this study displayed negligible cytotoxicity against SK-N-SH cells, with the exception of ginsenoside Rd and clinafloxacin HCl, which exhibited cytotoxic effects at concentrations exceeding 200 µM and 150 µM, respectively. Interestingly,certain compounds appeared to stimulate SK-N-SH growth at specific concentrations (Fig. 2).

### Verification of candidate Drugs

**Fig. 3:**
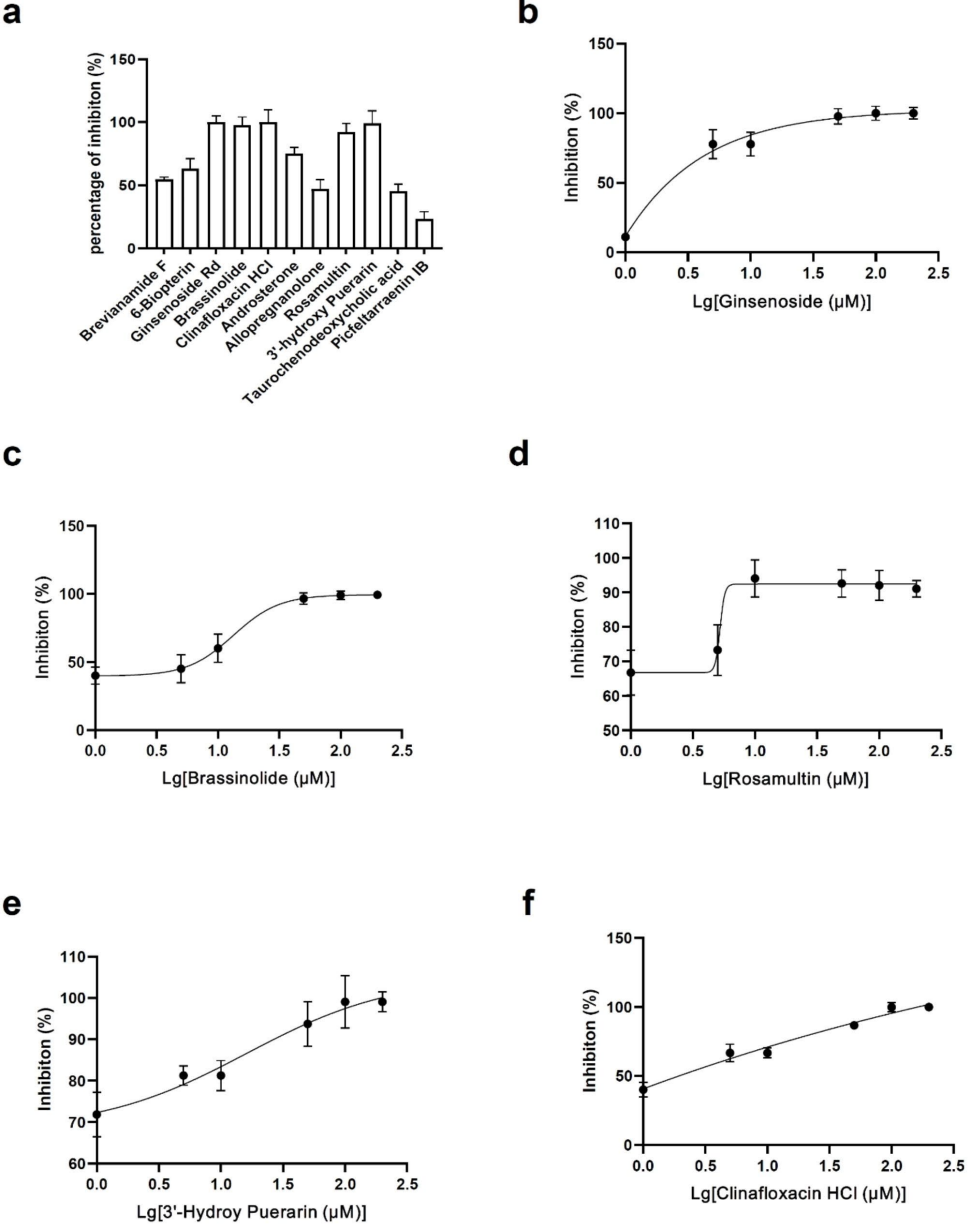
Percent inhibition of 11 candidate inhibitors against HSV-1 and dose-response effects of five inhibitors(a) Percent inhibition at the concentration of 100 µM. This is based on results of plaque reduction assay. This concentration is also used in first-round screening. (b-g) Dose-response curves of five inhibitors. A log transformation of these concentrations has been conducted

To verify the antiviral activity of the 11 candidate drugs that were identified by our screening assay, we conducted the plaque reduction assay, which is widely regarded as the gold standard method in virology. Ginsenoside Rd, brassinolide, rosamultin, 3’-hydroxy puerarin and clinafloxacin HCl significantly inhibited virus production at the concentration of 100 µM (Fig. 3a). The calculated selectivity index (SI, CC_50_/IC_50_) were displayed in Fig. 1c. The plaque reduction assay of brassinolide was shown in Fig.S1.

**Fig. 4:**
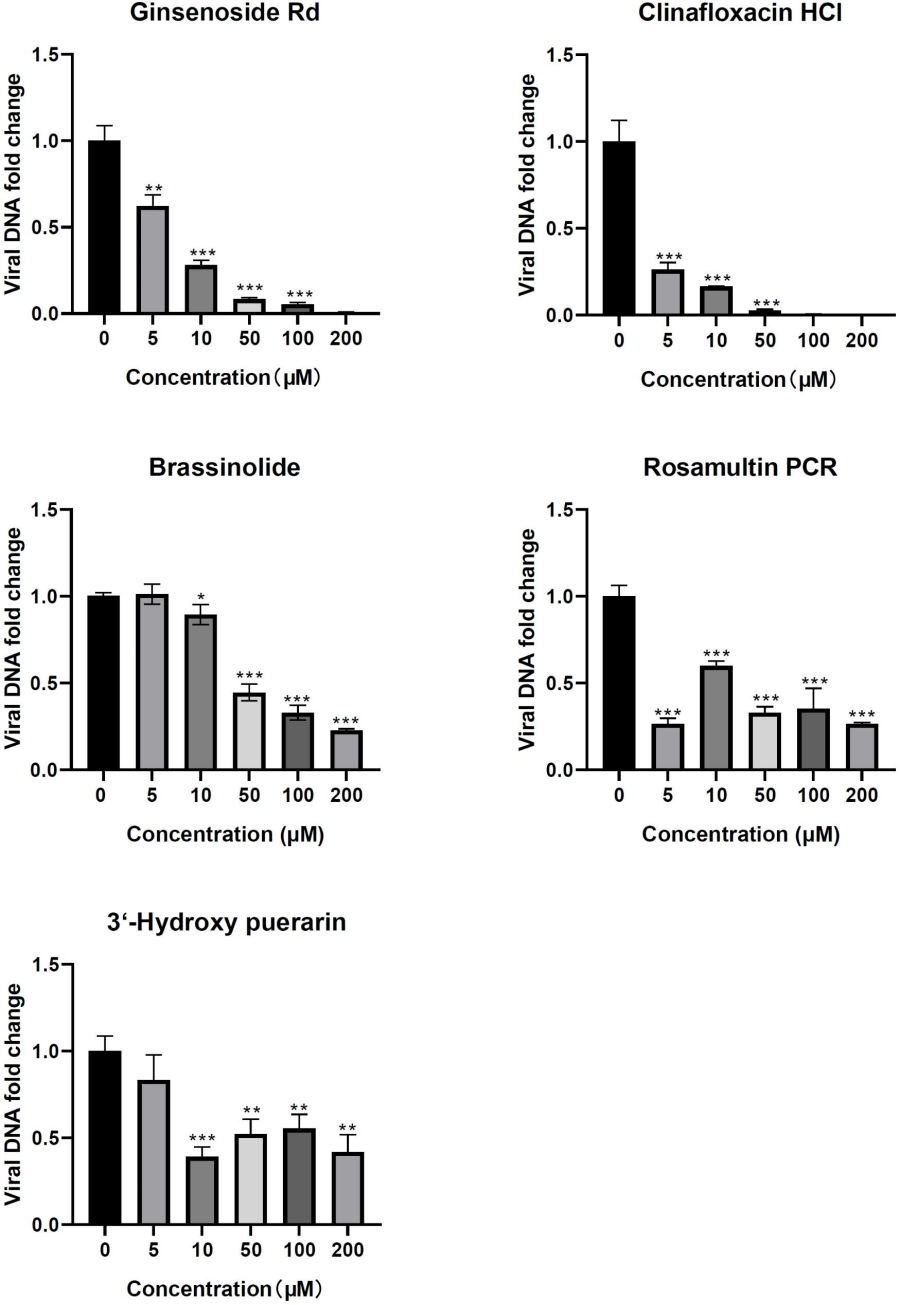
Inhibitory effects by relatively quantifying viral DNA in infected SK-N-SH cells. Data are presented as mean + SD. *P < 0.05, **P < 0.01, ***P < 0.001 vs. ‘o’ group

To investigate the compounds’ inhibitory effects on viral replication, relative quantification of viral DNA was performed. The results demonstrated that these compounds can dose-dependently reduce HSV-1 DNA, with ginsenoside Rd and Clinafloxacin HCl exhibiting significant inhibitory effects at a concentration of 5 µM (Fig. 4).

## Discussion

The current treatment utilizing nucleoside analogs that interfere with viral polymerase is indeed effective at eliminating a sensitive HSV strain in patients with acute infections. However, these drugs are ineffective for latent infections due to their poor availability to the nervous system. A promising drug candidate, IM-250, a helicase-primase inhibitor with sufficient exposure to the target tissue, has been reported to be effective against latent neural HSV infections [19, 20]. Nevertheless, this novel anti-HSV inhibitor has yet to be approved for clinical use.

The 11 lead drug candidates identified in this study are from natural sources with the exception of clinafloxacin HCl, a synthetic antibiotic. These drugs exhibit a wide range of bioactivities, such as anti-inflammatory, anti-tumor and antibacterial effects. One of the most important inclusion criteria was that the drugs had not been reported to have anti-HSV-1 activity in published literature. Some may have been reported in patents, but there are no practical examples.

Brassinolide is a plant hormone well-known for its versatile roles in promoting cell elongation, plant growth, seed germination, and responses to stress [21]. Epibrassinolide, which differs slightly from brassinolide in molecular conformation, has proven to be pro-apoptotic to cancer cells through the Wnt signaling pathway [22]. Our results showed that brassinolide can significantly suppress the HSV-1 proliferation in SN-N-SH cells, which was confirmed by plaque reduction assay and real time PCR. In general, this is consistent with the description of two published patents [23, 24].

Ginsenoside is a compound isolated from the root of Panax ginseng Meyer, a traditional herbal medicine used in East Asian countries. Ginsenoside 20(R)- Rh2 was reported to possess inhibitory effects on the replication of mice and human gammaherpesviruses [25]. Ginsenoside 20(S)-Rg3 demonstrated inhibitory effects on both HSV-1 and HSV-2 [26]. Ginsenoside Rb1 could inhibit nerve cell apoptosis caused by HSV-1 infections. Ginsenoside Rd is also a multi-functional natural compound, effective in neurologic disorders, cardiovascular diseases and tumors [27–30]. This type of ginsenoside has not been reported to have significant antiviral activity against any virus [31]. In our experiment, Ginsenoside Rd can significantly inhibit the HSV-1 at a low concentration(IC50 = 3.0, SI > 66.67)in vitro, although it showed obvious cytotoxicity against SK-N-SH cells at concentrations higher than 200 µM.

Rosamultin, isolated from Rosa rugose root, has been reported to possess anti-hepatotoxic effects, protective effects against H_2_O_2_-induced oxidative stress, and anti-apoptosis in cardiomyocytes [32, 33]. Additionally, it was reported that rosamultin exhibits antiviral activity by inhibiting HIV-1 protease in vitro [34]. 3’-hydroxy puerarin, one of isoflavones isolated from the flowers of Pueraria lobate, has been identified as an inhibitor of lactate dehydrogenase [35]. Our study demonstrated that both compounds can inhibit HSV-1 at high concentrations, although no antiviral activity of these plant-derived compounds has been reported previously. Picfeltarraenin IB, one of cucurbitacins isolated from picria fel-terrae, showed no antiviral activity against HSV [36]. However, our results indicate that it can slightly inhibit the replication of HSV-1 in SK-N-SH cells at the concentration of 100 µM (Fig. 3).

As a member of the fourth generation fluoroquinolones with broad antibacterial activity, clinafloxacin can inhibit both gram-negative and gram-positive bacteria by targeting DNA gyrase or topoisomerase [37]. To date, although fluoroquinolones such as enoxacin, ciprofloxacin, levofloxacin, and moxifloxacin have been reported to exhibit low antiviral activity against SARS-CoV-2 and MERS-CoV in vitro, no literature has shown that clinafloxacin has antiviral effects [38]. In our study, clinafloxacin HCl, with much higher solubility than clinafloxacin itself, demonstrated significant anti-HSV-1 activity in vitro, although its antiviral mechanism remains unclear.

Brevianamide F, a tryprostatin-type compound isolated from actinomycete, is a potential natural plant growth inhibitor or a broad-spectrum systemic herbicide [39]. On the other hand, brevianamide F has shown good anti-BCG (tuberculosis) activity [40].

6-biopterin, a cofactor of NO synthase, is an oxidized product of (6R)5,6,7,8 tetrahydrobiopterin (6-BH4) and exhibits extreme cytotoxicity to human melanocytes [41]. In our cytotoxicity test, 6-biopterin seems to be able to promote SK-N-SH cell growth in vitro (Fig. 2). On the other hand, it can suppress HSV-1 growth in host cells (Fig. 3).

Taurochenodeoxycholic acid (TCDCA), a bioactive substance of animal bile produced by the liver, acts as an agonist of farnesoid X receptor (FXR) in the digestive system [42, 43]. While it has been proposed as a compound for treating enveloped viral infections such as influenza, parainfluenza ,human immunodeficiency, and herpes viruses, no antiviral activity of this compound has been reported against HSV-1 so far [44]. Allopregnanolone, an endogenous reproductive neurosteroid, plays a vital role in controlling inflammatory processes and behavior. Owing to its neuroprotective effect, allopregnanolone also plays an important role in promoting fetal brain development [45]. It can affect the inflammatory reaction and repair alveolar respiratory epithelium damaged by influenza virus infection [46]. Epiandrosterone (EA) and Dehydroepiandrosterone (DHEA) demonstrated in vitro antiviral activity against Junin virus(JUNV)and adenovirus (AdV) replication by inhibiting protein synthesis [47, 48].

In a previous study, a screening method was employed to discover anti-EV71 inhibitors from a small library, and several candidate drugs belonging to flavonoids were identified in the final [16]. This screening strategy does not target any specific part of viruses or host cells, allowing for the inclusion of almost all kinds of antiviral compounds in screened libraries. While this may increase the success rate of discovering effective antiviral drugs, it may also result in a lager workload for efficacy verification and mechanism research, particularly when numerous candidate drugs are identified after the final screening round.

The plaque inhibition test was performed to screen for potential inhibitors of HSV-1 infection. A total of 88 compounds were identified as candidates at a concentration of 100 µM, which completely blocked the viral lifecycle and prevented the formation of plaques on Vero cell monolayers by single infectious HSV-1 particles. This concentration was chosen to simplify the interpretation of results by using a binary criterion (presence or absence of plaques). A dose-dependent reduction of plaque size would be expected in a plaque inhibitory test, but it was not assessed in this screening round.

We have identified 11 potential inhibitors of HSV-1 infection by using an improved plaque inhibition test in a two-round screening process. Five of these inhibitors were confirmed by two independent methods in a short time. These inhibitors may have novel mechanisms of action that are not yet known, or they may have existing antiviral effects that have not been reported before. To further explore the mode of action of these inhibitors, we plan to conduct more experiments to investigate how they affect different stages of the viral replication cycle, such as attachment, entry, uncoating, genome replication, assembly, and release. This will help us understand the molecular basis of their antiviral activity and optimize their therapeutic potential.

### Electronic supplementary material

Below is the link to the electronic supplementary material.

Supplementary Material 1

Supplementary Material 2

## Data Availability

All data generated or analyzed during this study are included in this published article and its supplementary information files.

## Abbreviations

HSV-1: Herpes simplex virus type 1
qPCR: Real time polymerase chain reaction
MOI: Multiplicity of infection
CC_50_: The 50% cytotoxic concentration
IC_50_: The half maximal inhibitory concentration
