# Supplementary material for: Screening and verification of antiviral compounds against HSV-1 using a method based on a plaque inhibition assay
Source: BMC Infect Dis. 2023 Dec 19;23:890. doi: 10.1186/s12879-023-08843-3 (PMC10731695; doi:10.1186/s12879-023-08843-3)
Supplement: Supplementary file 2 — Supplementary Material 2 [file 12879_2023_8843_MOESM2_ESM.docx]

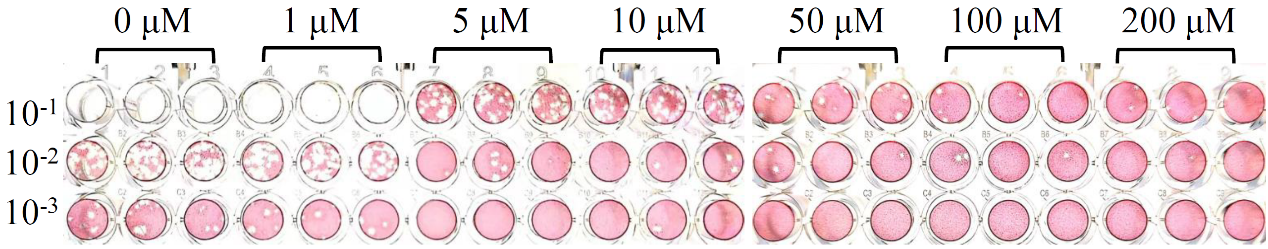


**Fig.S1** Plaque reduction assay of brassinoside against HSV-1. The supernatants of HSV-1 infected SK-N-SH cells were transferred to test the viral titers on Vero cell monolayers in 96-well plates. This is only one example of five inhibitors.
